# Latent factors affecting safer injection practices that can reduce infections and how education can improve them

**DOI:** 10.1371/journal.pone.0308567

**Published:** 2024-10-18

**Authors:** Jung Wan Park, Samel Park, Eunjung Lee, Tark Kim, Eu Suk Kim, Bongyoung Kim, So Yeon Yoo, Su Ha Han, Tae Hyong Kim

**Affiliations:** 1 Division of Infectious Diseases, Department of Internal Medicine, Soonchunhyang University Cheonan Hospital, Soonchunhyang University College of Medicine, Cheonan, Republic of Korea; 2 Division of Nephrology, Department of Internal Medicine, Soonchunhyang University Cheonan Hospital, Soonchunhyang University College of Medicine, Cheonan, Republic of Korea; 3 Division of Infectious Diseases, Department of Internal Medicine, Soonchunhyang University Seoul Hospital, Soonchunhyang University College of Medicine, Seoul, Republic of Korea; 4 Division of Infectious Diseases, Department of Internal Medicine, Soonchunhyang University Bucheon Hospital, Soonchunhyang University College of Medicine, Bucheon, Republic of Korea; 5 Division of Infectious Diseases, Department of Internal Medicine, Seoul National University Bundang Hospital, Seoul National University College of Medicine, Seongnam, Republic of Korea; 6 Department of Internal Medicine, Hanyang University College of Medicine, Seoul, Republic of Korea; 7 Nursing College, Gachon University, Seongnam, Republic of Korea; 8 Department of Nursing, Soonchunhyang University, Asan, Republic of Korea; Wolkite University, ETHIOPIA

## Abstract

**Background:**

The incidence of healthcare-associated infections, particularly injection-related infections, can increase patient comorbidities even in countries with adequate medical resources. Although there are clear guidelines for injection practices to prevent infections, their application in clinical settings is insufficient. Therefore, the objective of this study was to identify factors affecting injection practices associated with reduced infections by conducting surveys targeting practicing healthcare providers involved in administering injections at each healthcare organization and performing data analysis.

**Methods:**

We administered a survey to healthcare providers responsible for injection practices at each healthcare organization that included items related to infection-safe injection practice guidelines. All survey questions were reviewed by an expert panel of infectious disease and infection control nurses. Survey contents were subjected to exploratory factor analysis (EFA), confirmatory factor analysis, and multivariable robust regression tests to determine the impact of each factor and their correlations.

**Results:**

A total of 842 questionnaires were analyzed. Each questionnaire was classified into four factors: reuse and contamination, compliance with aseptic technique, exchange of infusion set, and use of multidose vials. Nurses with higher careers showed more compliance. Education within one year and awareness of each item of the questionnaire had positive associations with proper injection practice.

**Conclusions:**

Education is thought to be the most important factor in good injection practices that could reduce infections. Relevant knowledge through timely training is expected to have a positive impact on performance and compliance related to safe injections.

## Introduction

Healthcare-associated infections can increase patient mortality, hospital stay, and consumption of medical resources even in developed countries, although they are preventable [1]. Among various procedures performed in hospitals, the most commonly performed procedures are intravenous (IV) and intramuscular (IM) injections, which should strictly follow standard precautions because they could lead to infiltration of pathogens, often resulting in large in-hospital outbreaks when procedures are performed inadequately [2]. Since intravenous injection is the most frequently performed invasive procedure in medical institutions and an essential treatment for patients, improper practice can culminate in several adverse effects. A surveillance study in 2004 reported that 24,179 cases of nosocomial bloodstream infections occurred over seven years [3]. According to a 2010 study where researchers investigated 20 ICUs for the incidence of bloodstream infection associated with a centrally inserted catheter, about 3.3 cases per 1,000 central line-days were confirmed [4]. There are several reports on infections due to inappropriate injection practices, including an outbreak of bloodstream infection caused by contaminated propofol during anesthesia [5] and hepatitis B or C outbreak caused by a dispensing process leading to exposure to disease-causing pathogens [6–8]. In Korea, HCV outbreaks have been reported to be caused by reuse of disposable syringes [9]. Non-tuberculosis Mycobacterium (NTM) skin and soft tissue infection of buttocks due to contamination during IM injection have been reported [10].

A survey on injection safety for injection prevention conducted by Korean National Evidence-based Healthcare Collaborating Agency (NECA) has reported that injection training, occupational status, and in-hospital culture on patient safety can affect injection safety [11]. Injection training can reduce errors in actual injection practice. Occupational status reflects the familiarity of techniques according to the accumulation of experience. Cultural factors can be used to estimate the degree of infection prevention practices in hospitals. Although the Korea Disease Control and Prevention Agency and Korean Society for Healthcare-associated Infection Control published a standard prevention guideline for healthcare-associated infections in 2017, which includes safe injection practice [11], the practice for infection prevention during injection procedures seemed to be not well-followed.

Although many infection prevention guidelines are already in place for injection practices, injectable infections are still a problem. We wondered if this was due to barriers to practicing injections in clinical settings. Thus, we conducted a survey of nurses in charge of injection practice in real-world practice to evaluate factors affecting the performance and awareness of adherence to and obstacles to conform to guidelines on injection practice. In particular, we conducted an exploratory factor analysis (EFA) to determine whether behaviors or circumstances corresponding to each survey question could serve as potential variables affecting injection procedures associated with reduced infections.

## Methods

### Study design

This study performed a descriptive survey of healthcare workers involved in injection practices to determine their awareness, awareness, safety climate, and performance of standardization and to identify factors affecting their performance. In addition to providing a comprehensive overview of current practices, this study also performed exploratory factor analysis (EFA) to identify underlying factors affecting healthcare providers’ adherence to injection protocols associated with reduced infections. The central research question is: ’What are the key factors influencing healthcare providers’ compliance with infection-safe injection practices?‴

### Creation of the questionnaire

Experts who were in charge of the practice of infection prevention during injection were grouped to build questionnaire items. The group consisted of six doctors specializing in infectious diseases, three nurses with expertise in infection control, and two nursing professors. We examined various pre-existing guidelines related to injection practice, including standard prevention guidelines for hospital-associated infections [11], analysis of domestic injection safety management by the National Evidence-based Healthcare Collaborating Agency (NECA) [12], Food and Drug Administration’s Guidelines for Safe Use of Injectables [13], infusion therapy standards of practice [14], and Association for Professionals in Infection Control and Epidemiology (APIC) position paper (including safe injection, infusion, and medication vial practices in health care) [15]. We produced a questionnaire by combining and integrating questions that arose from an evidence-based safe injection practice described above. Contents of the survey questionnaire were organized to evaluate strategies frequently missed during injection practices and reasons why they were missed. In addition, missing patterns that were performed unconsciously, causes of non-adherence to recommended guideline instructions, and obstacles to compliance with guidelines for injection safety were purposes of the questionnaire.

The questionnaire consisted of two parts. The first part consisted of 27 items. Each item was designed to examine whether participants knew about precautionary strategies (i.e., awareness) with response of yes or no and how well they followed these strategies to prevent infection during injection procedures (i.e., performance). The degree of adherence to these strategies was answered using a five-point Likert scale (from 1 to 5: 1, never, 2: rarely, 3: sometimes, 4: usually, and 5: always). In the second part of the questionnaire, questions regarding obstacles when following strategies were formed with ten items: hindrance (response to yes or no).

### Validation

We consulted 10 infectious disease experts, including infectious disease physicians and infection control nurses, to determine content validity of survey questions. As a result, 96 out of 106 questions had a content validity index (CVI) of 0.8 or higher. These questions were used in the survey.

### Sampling and data collection

We planned to include as many hospitals as possible and survey small, medium-sized, and tertiary hospitals. We commissioned medical institutions where researchers were working to recruit subjects for the survey. We contacted various types of medical institutions through the Infection Control Consulting Network (ICCON). We selected medical staff directly in charge of injection practice at each medical institution as subjects of the questionnaire. All surveys were conducted online using SurveyMonkey^®^ over a two-month period from December 2020 to January 2021, strictly adhering to data privacy and security regulations. We excluded duplicate answers and incomplete answers from the analysis target when analyzing questionnaire items.

### Sample size determination procedure

To the best of our knowledge, there was no known method that could determine the sample size a priori in the exploratory factor analysis (EFA). A previous work has shown that a sample size of more than 50 people is sufficient to perform EFA. We have about 800 participants. Thus, we believed that this sample size was a sufficient to perform EFA [16, 17].

### Sampling techniques

We did not have a specific sampling technique. Rather, several professors of infectious diseases and nursing came together to develop a consensus and conducted a survey of hospitals that participated in the project as a national project. Participation in the survey was voluntary on an individual basis.

### Inclusion and exclusion criteria

Inclusion criteria were: healthcare workers responsible for actual injection practices. Participants were drawn from four large medical institutions with more than 800 beds in Seoul, Gyeonggi-do, and Chungcheongnam-do. Additionally, healthcare workers from small and medium-sized hospitals (fewer than 200 beds) affiliated with the Infection Control Consulting Network (ICCON) were included. All participants were required to consent to participate in this study and to complete the survey. During the data cleaning process, exclusion criteria were applied to remove incomplete or duplicate responses, thereby ensuring data integrity and quality.

### Statistical analysis

Several statistical methods can reduce the dimensionality of large interrelated variables to increase data interpretability. A principal component analysis, one of the methods to reduce dimensionality, can be performed to reduce and find principal components from many variables. We have previously reported principal components associated with death in patients using pesticide components [18]. Meanwhile, a factor analysis, including exploratory factor analysis (EFA) and confirmatory factor analysis [19], is a well-known statistical method in the field of psychology to extract latent variables (namely factors) from surveillance data [20].

An exploratory factor analysis was used to identify latent variables that could impede the prevention of aseptic injections. In addition, it could help us understand latent behavior patterns during injection procedures. A scree plot and parallel test were used to calculate adequate number of factors. Promax rotation was used to calculate factor loading using the maximal likelihood method. Factors with loading > 0.2 are presented in result tables. Multivariable robust regression tests were performed to evaluate factors affecting latent variables. We used robust regression, not a linear regression model, because dependent variables were not normally distributed. Based on the scree plot and parallel test, the adequate number of factors to represent the performance of responders was found to be four, leading to four tests to estimate the association between each factor and covariates. To avoid false positives that might occur through multiple comparisons, *P*-value was adjusted using Bonferroni correction.

### Ethics statement

This study was approved by the Institutional Review Board (IRB) of Soonchunhyang University Hospital, Seoul, Korea (IRB No. 2020-07-034). The requirement for informed consent was waived by the IRB since only anonymized data were collected.

## Result

### Survey participants

A total of 1,105 nurses participated in this survey. Among them, 200 participants were excluded because of missing values in the questionnaire. A total of 62 respondents were excluded because they did not perform injections during the previous year. One respondent was excluded because the workplace was a dental department. Finally, 842 respondents were eligible for further analysis (S1 Fig).

Table 1 shows baseline characteristics of participants. About 70% of participants were working at a tertiary hospital and 78% were working at hospitals in the Seoul metropolitan area. A total of 36% and 49% of respondents worked at medium-sized (500–999 beds) and large-sized (≥ 1000 beds) hospitals, respectively. In addition, 68% were general nurses, about 20% were executive nurses at a head nurse level, 65% were working in general wards, and 12% were working in special wards such as ICU or ER. More than 80% of participants answered that they had received training related to injection practice within one year. Among them, 63.2% had participated online. Ninety-six percent of respondents answered that there was an in-hospital guideline or an administrative and educative department in charge of training during injection practice.

**Table 1:** Baseline characteristics of participants (N = 842).

List of survey items	N (%)
**Hospital type**	
Mid-tier hospitals or long-term care facilities	108 (12.8)
General hospitals	139 (16.5)
Tertiary general hospitals	595 (70.7)
**Area**	
Seoul metropolitan area	659 (78.3)
Other area	183 (21.7)
**Hospital size, beds**	
<500	123 (14.6)
500–999	303 (36.0)
≥1000	416 (49.4)
**Department**	
General ward	543 (64.5)
ICU, NICU, and ER	184 (21.9)
Other departments^a^	115 (13.7)
**Grade**	
General nurse	568 (67.5)
Middle-management (charged nurse)	101 (12.0)
Executive (headed nurse)	173 (20.5)
**Age** ^b^	33.5 ± 9.1^c^
< 30	386 (45.8)
30–39	226 (26.8)
≥ 40	230 (27.3)
**Female**	824 (97.9)
**Have been educated within 1 year on injection-practice within a year, yes**	676 (80.3)
Online	427 (63.2)
Offline	249 (36.8)
**The presence of an administrative and educative department in the hospital, yes**	809 (96.1)
**Career**	
< 3 years	217 (25.8)
3–10 years	274 (32.5)
≥ 10 years	351 (41.7)

^a^ Other departments included operating rooms, outpatient clinics, endoscopy wards, anesthesia recovery wards, delivery rooms, intervention rooms, hemodialysis units, and outpatient injection units.

^b^ Two participants refused to answer their age.

^c^ Age was represented as mean ± standard deviation.

**Abbreviations:** ICU, intensive care unit; NICU, neonatal intensive care unit; ER, emergency room.

### Raw results before factor analysis

S1 Table in S1 File shows results of each questionnaire divided by performance and awareness. The mean value was above 4.5, implying that participants almost always followed infection prevention strategies. In addition, more than 95% of respondents answered yes to questionnaire items on awareness (except for an item stating that the fluid set for injecting propofol should be changed every injection or every six to 12 hours–only 91.7% of respondents answered yes), indicating that they knew these strategies.

S2 Table in S1 File shows obstacle factors (represented as hindrance) hampering proper injection procedures recommended by the guidelines. More than 50% of respondents answered yes to “There is not enough time to follow the injection practice guidelines for infection prevention, such as aseptic techniques, hand hygiene, and disinfection.” The next most frequently chosen cause was difficulty of the aseptic technique, with a response rate of 39.4%. Unfortunately, more than 20% of respondents replied that they lacked supplies, environment, and facilities for safe injection practices.

### Construction of latent variables using factor analysis

The 27 questions associated with performance scores were reduced and converted into four factors (i.e., latent variables) (Table 2). An adequate number of factors was calculated using the scree plot and parallel test as described in the Methods section. These factors were named based on factor loadings, resulting in “Reuse and contamination,” “Compliance with aseptic technique,” “Exchange of infusion set,” and “Use of multidose vials.” The "Reuse and contamination" factor was about the degree of reuse of syringes, needles, injectable drugs, and so on or the use of injectable items that might be contaminated. "Compliance with aseptic techniques " was related to compliance with aseptic techniques such as hand hygiene and disinfection during injection practice. "Use of multi-dose vials" was associated with precautions when using vials that could be used as multi-doses.

**Table 2:** Exploratory factor analysis (EFA) of awareness and performance factors.

Variables	Factor 1	Factor 2	Factor 3	Factor 4
Eigenvalue (SS* loadings)	4.52	3.3	2.35	1.2
Proportion of variance	0.17	0.12	0.09	0.04
Cumulative variance	0.17	0.29	0.38	0.42
Reuse and contamination				
Do not collect the remaining injection drugs in a separate container after use on one patient	0.97			
Do not transfer drugs from a syringe containing injection drugs to another syringe	0.87			
Do not remove the rubber stopper to use the drug in the vial.	0.74			
Do not use for flushing of multiple patients by drawing fluid from a sterile infusion bag or sterile infusion bottle.	0.72			
Do not carry syringes and needles containing injections in uniform pockets.	0.71	-0.2	0.23	
Do not reuse syringes or needles once connected to the infusion bag or infusion set.	0.67			
Even if the needle is changed, the syringe is not reused.	0.65			
Do not put the needle in the rubber stopper of the vial injection drug.	0.52	0.42		
Compliance with aseptic technique				
Prepared injection drug is administered within one hour.		0.72		
Wait for the disinfectant to dry out when disinfecting the skin before injection.		0.64		
Before handling intravenous injections, do hand hygiene before preparing injection drugs.		0.6		
Sterilized syringes and needles are opened immediately before use.		0.58		-0.2
Follow sterilization in the preparation and administration of injection drugs.		0.57		
Before and after use, the injection port is thoroughly wiped with disinfectant for three to 15 seconds and dried.		0.54		
If the package is opened or damaged, even an unused syringe is considered contaminated and discarded.		0.51		
Injectable drugs that cannot be administered immediately should be labeled on the syringe with the ingredients, dosage, and date and time of preparation.		0.41		
Do not use the opened syringe even if there is no visible contamination.	0.22	0.37		
When removing drugs using a syringe, the rubber stopper of the vial must be sterilized with a disinfectant such as alcohol.		0.33		
Exchange of infusion set				
The TPN** infusion set is exchanged every time a new fluid is connected or every 24 hours.			0.86	
The infusion set for injecting drugs intermittently is exchanged every injection or every 24 hours.			0.68	
If you must use a 3-way connector, attach a sterilization cap to the inlet to maintain a closed system.			0.65	0.22
The transfusion set and filters for injecting blood products should be changed every time the blood bag is changed or every 4 hours.			0.43	
The 3-way connector must be exchanged together when changing the infusion set.			0.38	
The fluid set for injecting propofol is changed every injection or every 6 to 12 hours.		0.22	0.31	
Single-use vials are for one patient only, and the remaining medication is discarded.			0.23	
Use of multi-dose vials				
The expiration date of the multi-dose vial is recorded when first opened.				0.73
Opened multi-dose vials should be discarded within 28 days after opening unless the manufacturer has a specific expiration date.			0.22	0.55

* SS loading; sum of squared loading,

** TPN; Total Parenteral Nutrition

An adequate number of factors was determined to be four based on the scree plot and parallel test as described in the Methods section. Factor loadings were calculated using maximal likelihood methods and then recalibrated using the Promax rotation. Factors with loading > 0.2 are presented in this table. Results were ordered according to factor loading. Each factor was named based on values of factor loadings of the questionnaire as follows: Factor 1, "Reuse and contamination"; Factor 2, "Compliance with aseptic technique"; Factor 3, "Exchange of infusion set"; and Factor 4, "Use of multi-dose vials."

In Table 3, obstacle factors that disturbed proper injection practice were reduced into two factors: 1) “External factors” originated from the in-hospital environment; and 2) “Internal factors” caused by individual characteristics.

**Table 3:** Exploratory factor analysis (EFA) of obstacle factors.

Variables	Factor 1	Factor 2
Eigenvalue (SS loadings)	3.53	2.46
Proportion of variance	0.35	0.25
Cumulative variance	0.35	0.6
External factors		
There are no practical guidelines for injections to prevent infection in the working medical institution	1.11	-0.23
Lack of opportunities for training on injection practice for infection prevention	0.91	
It is difficult to understand the contents of the injection practice guidelines for infection prevention in working medical institutions	0.75	
There is no penalty or penalty for non-compliance with the injection practice guidelines	0.65	
There is no organizational culture that complies with the injection practice guidelines for infection prevention	0.53	0.33
Internal factors		
There is not enough time to follow injection practice guidelines for infection prevention, such as aseptic technique, hand hygiene, and disinfection.	-0.31	1.13
It is difficult to comply with aseptic technique in all processes, such as preparation and administration of injectable drugs.		0.82
I think it is a waste to use only a portion of the injection and throw it away.		0.44
The environment and facilities are not in place to safely comply with the injection practice guidelines for infection prevention.	0.36	0.43
Insufficient supplies such as syringes, needles, and infusion set for safe use	0.29	0.29

An adequate number of factors was decided to be two based on the scree plot and parallel test as described in the Methods section. Factor loadings were calculated using maximal likelihood methods and then recalibrated using the Promax rotation. Factors with loading > 0.2 are presented in this table. Results were ordered according to factor loading. Each factor was named based on values of factor loadings of the questionnaire as follows: Factor 1, " external factors”; and Factor 2, “internal factors.”

### Factors affecting awareness and performance

Next, we performed a multivariable robust regression test to investigate factors affecting the performance of proper injection practice, denoted as “Reuse and contamination,” “Compliance with aseptic technique,” “Exchange of infusion set,” and “Use of multi-dose vials (Table 4).” Values in Table 4 show β estimates of the robust regression test, in which positive values mean variables could affect performance factors positively. Asterisks denote p-values. Hospital location did not affect any performance factors. Medium-sized hospitals with 500–999 beds had the most positive association with “Compliance with aseptic techniques” (β = 0.200; *P* < 0.05). Special departments, including ICU, NICU, and ER, showed more compliance with performance factors except for “Use of multi-dose vials.” As expected, nurses with higher careers showed more compliance in “Reuse and contamination” and “Compliance with aseptic technique” (in nurses with careers ≥ 10 years, β estimates were 0.082 and 0.091, respectively). Education within one year showed significant positive associations with “Compliance with aseptic technique”, “Exchange of infusion set”, and “Use of multi-dose vials” (β estimates were 0.040, 0.079, and 0.022, respectively). Interestingly, there were no differences in results between online and offline educations. Awareness of each item of the questionnaire had positive associations with proper injection practice. The external obstacle factor was associated with “Use of multi-dose vials,” and the internal factor was related to others, i.e., “Reuse and contamination,” “Compliance with aseptic technique,” and “Exchange of infusion set.”

**Table 4:** Factors affecting the performance of proper injection practice.

	Performance factors			
Variables	Reuse and contamination	Compliance with aseptic technique	Exchange of infusion set	Use of multi-dose vials
**Area**				
Seoul metropolitan area	Ref.	Ref.	Ref.	Ref.
Other area	-0.010	-0.004	-0.047	0.021
**Hospital size, beds**				
< 500	Ref.	Ref.	Ref.	Ref.
500–999	0.038	0.200***	0.154	0.037
≥ 1000	-0.046	0.108	-0.228	0.053
**Department**				
General ward	Ref.	Ref.	Ref.	Ref.
ICU, NICU, and ER	0.049*	0.108**	0.165*	-0.058*
Other department^a^	0.040	0.092*	0.178*	-0.074**
**Grade**				
General nurse	Ref.	Ref.	Ref.	Ref.
Middle-management (charged nurse)	0.011	-0.012	0.044	-0.032
Executive (headed nurse)	0.022	0.031	0.081	-0.015
**Career**				
< 3 years	Ref.	Ref.	Ref.	Ref.
3–10 years	0.051*	0.061	0.067	0.013
≥ 10 years	0.082***	0.091*	0.155	0.013
**Have been educated on injection-practice within a year** ^b^				
No vs. Yes	0.014	0.040***	0.079***	0.022**
Online vs. Offline	0.007	0.004	-0.008	0.007
**The presence of an administrative and educative department in the hospital, yes**				
No	Ref.	Ref.	Ref.	Ref.
Yes	-0.050	-0.109	-0.012	0.040
**Awareness factors** ^c^				
Reuse and contamination	0.229***	0.056	0.189*	-0.015
Compliance with aseptic technique	0.022	0.307***	0.181***	-0.022
Exchange of infusion set	0.019	0.047	0.278***	0.073***
Use of multi-dose vials	0.020	0.040	0.078	1.019***
**Obstacle factors** ^d^				
External factors	-0.009	-0.010	0.037	-0.035**
Internal factors	-0.049***	-0.078***	-0.258***	-0.010

A multivariate robust regression test was performed. B-coefficient values are presented. P-values were calculated after Bonferroni correction.

^a^ Other departments included operating rooms, outpatient clinics, endoscopy wards, anesthesia recovery wards, delivery rooms, intervention rooms, hemodialysis units, and outpatient injection units.

^b^ Planned contrasts were used. At first, cases with education within a year were compared to cases without. Cases with online education were then compared to cases with offline education.

^c^ Awareness factors were calculated as 1 point for answering yes to each questionnaire based on awareness and performance factor results (Table 2). Therefore, the maximum scores for “Reuse and contamination”, “Compliance with aseptic technique”, “Exchange of infusion set”, and “Use of multidose vials” were 8, 10, 7, and 2, respectively.

^d^ The value of the obstacle factor is the factor loading calculated based on the questionnaire (Table 3).

*, *P*-value < 0.05;

**, *P-*value < 0.01;

***, *P*-value < 0.001.

Ref.: A regression analysis against the reference in a multivariate robust regression test was performed to determine how much it affected the reference.

**Abbreviations:** ICU, intensive care unit; NICU, neonatal intensive care unit; ER, emergency room.

## Discussion

This study identified factors affecting proper injection practices. These results were from nurses who performed injection procedures in participant medical institutions, including tertiary hospitals and smaller hospitals in the Republic of Korea. In addition, we extrapolated latent variables that could not be exposed using EFA, showing that cultivating awareness, represented as awareness in this study, and education on proper injection practice had a significant impact on compliance with safety injection guidelines of medical staff.

Several previous studies have been conducted to evaluate the behavior and comprehension of medical staff regarding the importance of safe injection practices. In a survey conducted on syringe and needle reuse among medical staff in a national injection safety campaign in the United States in 2021, 12% of doctors and 3% of nurses had inappropriately reused syringe. In 5% of cases, inappropriate practices were repeated [21]. A survey conducted to evaluate any difficulties during injection practice showed decreased compliance regarding needleless access devices (33%) and multi-dose vials (< 80%). Respondents also stated that they were too busy and understaffed to perform proper infection-prevention strategies [22], concordant with our observation. In a previous cross-sectional study conducted in Korea, factors affecting the performance of proper intravenous injection practice of nurses in small and medium-sized hospitals were awareness of the importance of the in-hospital system for infection control and awareness of preventive strategies for safe injection [23]. In our survey, more than 90% of respondents answered that they were aware of each question. Thus, there was no difference in the degree of awareness. However, good awareness was strongly associated with good performance. The cultivation of appropriate awareness is definitely a strong motivation for good performance. The overall performance of nurses who received training on injection practice for infection prevention within one year was further increased.

The main difference between our study and previous findings was that we confirmed with greater certainty that healthcare workers were aware of guidelines for injection practices to prevent infection. However, they did not follow these guidelines despite their awareness. In addition, advanced statistical methods have been used to demonstrate the need for ongoing awareness through appropriate training. However, we do not believe these findings differ from previous studies regarding their fundamental direction or the need for major improvements.

Regarding online education, there was no difference in the performance of injection practice compared to the traditional offline education method (Table 4). While face-to-face education opportunities are greatly reduced due to the COVID-19 pandemic, newly emerging online education can be a good alternative to provide effective awareness related to injection techniques (Table 4). This study further revealed several potential latent variables in safe injection practice (Table 4). The administrative region to which the hospital located did not affect respondents’ actual performance. As for hospital size, compliance to safe injection precautions was better in medium-sized hospitals (500–999 beds). In fact, time conflict associated with higher work load among workers in larger hospital probably led to a decline in performance of certain factors regarding injection safety. External interfering factors of hospital’s infection prevention environment were: "Reuse and contamination,” "Compliance with aseptic technique,” and "Exchange of infusion set." Not surprisingly, nurses with higher careers showed more adherence to preventive strategies including "Reuse and contamination" and "Compliance with aseptic techniques”. However, there was no significant correlation with career duration in performance related to changing infusion set or using multi-vial, implying that more complex external factors were influenced other than personal expertise.

Nurses working in intensive care units were more likely to comply with preventive strategies than those working in the general ward although the performance for the "Use of multi-dose vials" was lower among nurses working in intensive care units.

Some participant institutions were unable to perform the survey because of the unexpected COVID-19 pandemic, especially hospitals with limited number of workers. Participant hospitals finally investigated were relatively resourceful tertiary or university hospitals (70.7%) or those located in the Seoul metropolitan area (78.3%) (Table 1).

To add details related to hindrance factors in analysis results, although medical personnel had sufficient awareness and awareness of safe injection practices to prevent infection, they did not have enough time to follow guidelines or they found it difficult to apply consistent aseptic technique. To solve this problem structurally, more workers are needed, especially nurses.

In conclusion, our study showed results of a deeper analysis of the questionnaire, revealing factors affecting adherence to infection-preventive strategies of injection practice. In particular, the most effective factor in injection practice for infection prevention is education. Relevant awareness through timely education can positively affect the performance of correct compliance to safe injections.

## Supporting information

S1 File (DOCX)

S1 Fig Flow chart depicting an enrollment of the survey results. (DOCX)

## Abbreviations

APIC: Association for Professionals in Infection Control and Epidemiology
EFA: exploratory factor analysis
ICCON: Infection Control Consulting Network
IM: intramuscular
IV: intravenous
NECA: National Evidence-based Healthcare Collaborating Agency
NTM: Non-Tuberculosis Mycobacteria
