# Supplementary material for: Latent factors affecting safer injection practices that can reduce infections and how education can improve them
Source: PLoS One. 2024 Oct 18;19(10):e0308567. doi: 10.1371/journal.pone.0308567 (PMC11488737; doi:10.1371/journal.pone.0308567)
Supplement: S1 File — (DOCX) [file pone.0308567.s001.docx]

**Supplemental Table 1. Questionnaire Survey – Survey answer for Awareness and performance (N = 842)**

| **Questionnaire** | **Performance^a^** | **Awareness^b^** |
| --- | --- | --- |
| Before handling intravenous injections, do hand hygiene before preparing injection drugs | 4.5 ± 0.7 | 832 (98.8) |
| Follow sterilization in the preparation and administration of injection drugs. | 4.6 ± 0.6 | 836 (99.3) |
| Do not use the opened syringe even if there is no visible contamination. | 4.5 ± 1.1 | 820 (97.4) |
| When removing drugs using a syringe, the rubber stopper of the vial must be sterilized with a disinfectant such as alcohol. | 4.8 ± 0.5 | 835 (99.2) |
| Before and after use, the injection port is thoroughly wiped with disinfectant for 3 to 15 seconds and dried | 4.5 ± 0.7 | 806 (95.7) |
| Single-use vials are for one patient only, and the remaining medication is discarded. | 4.9 ± 0.4 | 835 (99.2) |
| Do not reuse syringes or needles once connected to the infusion bag or infusion set. | 4.9 ± 0.6 | 838 (99.5) |
| Do not use for flushing of multiple patients by drawing fluid from a sterile infusion bag or sterile infusion bottle. | 4.7 ± 0.9 | 829 (98.5) |
| Do not transfer drugs from a syringe containing injection drugs to another syringe | 4.7 ± 0.8 | 829 (98.5) |
| Do not collect the remaining injection drugs in a separate container after use on one patient | 4.8 ± 0.7 | 840 (99.8) |
| Do not put the needle in the rubber stopper of the vial injection drug. | 4.5 ± 0.9 | 807 (95.8) |
| Prepared injection drug is administered within 1 hour. | 4.4 ± 0.8 | 805 (95.6) |
| Do not remove the rubber stopper to use the drug in the vial. | 4.8 ± 0.7 | 836 (99.3) |
| Even if the needle is changed, the syringe is not reused. | 4.8 ± 0.7 | 826 (98.1) |
| Sterilized syringes and needles are opened immediately before use. | 4.7 ± 0.6 | 832 (98.8) |
| Injectable drugs that cannot be administered immediately should be labeled on the syringe with the ingredients, dosage, and date and time of preparation. | 4.7 ± 0.7 | 832 (98.8) |
| If the package is opened or damaged, even an unused syringe is considered contaminated and discarded. | 4.7 ± 0.6 | 832 (98.8) |
| The expiration date of the multi-dose vial is recorded when first opened. | 4.9 ± 0.5 | 834 (99.0) |
| Opened multi-dose vials should be discarded within 28 days after opening unless the manufacturer has a specific expiration date. | 4.8 ± 0.5 | 828 (98.3) |
| Wait for the disinfectant to dry out when disinfecting the skin before injection | 4.4 ± 0.7 | 809 (96.1) |
| The TPN infusion set is exchanged every time a new fluid is connected or every 24 hours. | 4.9 ± 0.5 | 834 (99.0) |
| The infusion set for injecting drugs intermittently is exchanged every injection or every 24 hours. | 4.7 ± 0.7 | 810 (96.2) |
| The fluid set for injecting propofol is changed every injection or every 6 to 12 hours. | 4.2 ± 1.4 | 772 (91.7) |
| The transfusion set and filters for injecting blood products should be changed every time the blood bag is changed or every 4 hours. | 4.8 ± 0.7 | 827 (98.2) |
| If you must use a 3-way connector, attach a sterilization cap to the inlet to maintain a closed system. | 4.9 ± 0.4 | 836 (99.3) |
| The 3-way connector must be exchanged together when changing the infusion set. | 4.7 ± 0.7 | 822 (97.6) |
| Do not carry syringes and needles containing injections in uniform pockets. | 4.9 ± 0.6 | 832 (98.8) |

^a^ Mean ± standard deviation; ^b^ number (%)

**Supplemental Table 2. Questionnaire Survey - Survey Answers to Obstacles Factors (N = 842)**

| **Questionnaire** | **Answer to yes^a^** |
| --- | --- |
| There is not enough time to follow the injection practice guidelines for infection prevention, such as aseptic technique, hand hygiene, and disinfection. | 466 (55.3) |
| It is difficult to comply with aseptic technique in all processes, such as preparation and administration of injectable drugs. | 332 (39.4) |
| I think it's a waste to use only a portion of the injection and throw it away. | 80 (9.5) |
| Insufficient supplies such as syringes, needles, and infusion set for safe use | 179 (21.3) |
| The environment and facilities are not in place to safely comply with the injection practice guidelines for infection prevention. | 186 (22.1) |
| There is no organizational culture that complies with the injection practice guidelines for infection prevention | 101 (12.0) |
| It is difficult to understand the contents of the injection practice guidelines for infection prevention in working medical institutions | 37 (4.4) |
| There are no practical guidelines for injections to prevent infection in the working medical institution | 46 (5.5) |
| Lack of opportunities for training on injection practice for infection prevention | 138 (16.4) |
| There is no penalty or penalty for non-compliance with the injection practice guidelines | 227 (27) |

^a^ number (%)
