# Supplementary figures and images for: Latent factors affecting safer injection practices that can reduce infections and how education can improve them
Source: PLoS One. 2024 Oct 18;19(10):e0308567. doi: 10.1371/journal.pone.0308567 (PMC11488737; doi:10.1371/journal.pone.0308567)

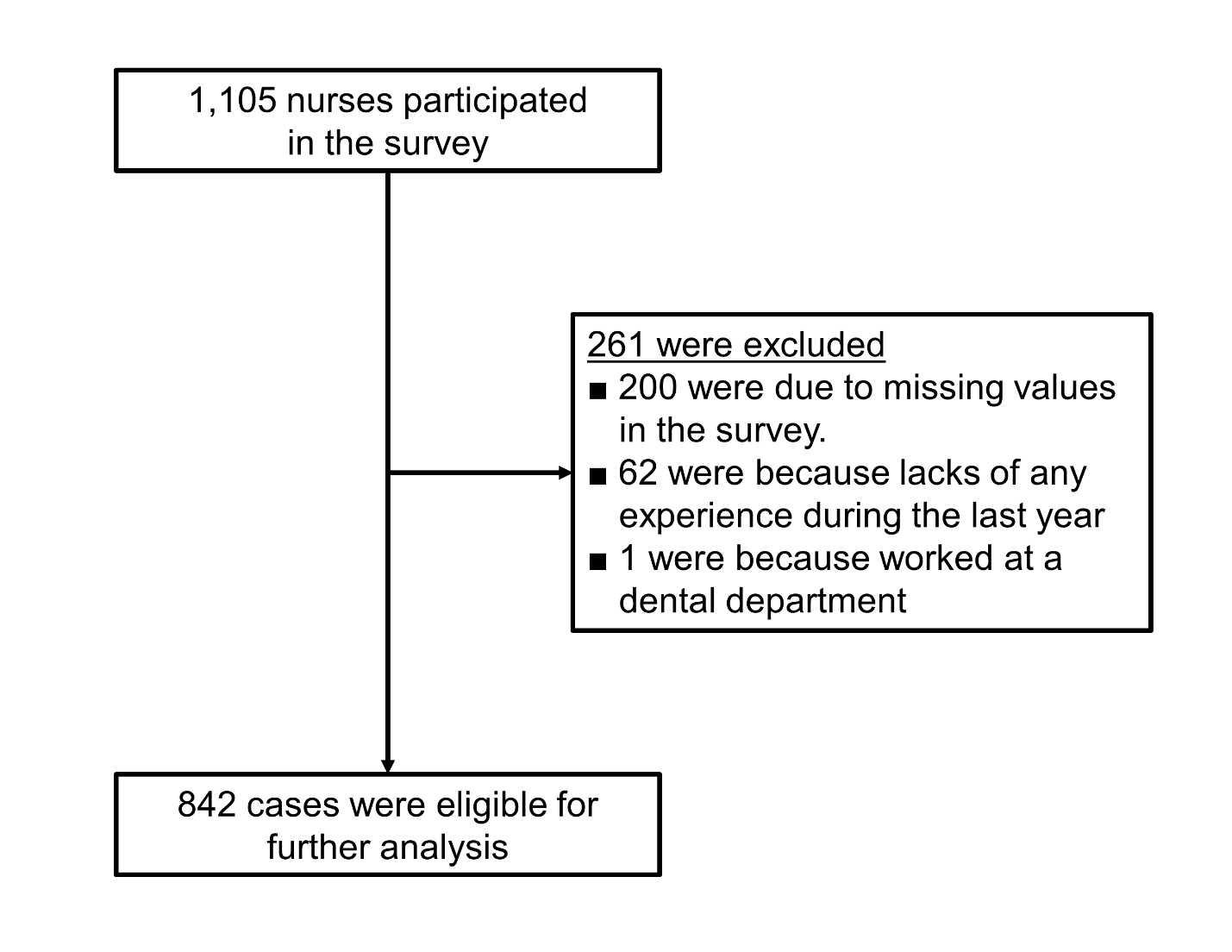


**Supplemental Figure 1.** Flow chart depicting an enrollment of the survey results

Supplement: S1 Fig — (DOCX) [file pone.0308567.s002.docx]
